# Cerebellar ataxia due to Leptospirosis- a case report

**DOI:** 10.1186/s12879-016-2081-2

**Published:** 2016-12-12

**Authors:** Rajveer Singh, Dheeraj Khurana, Sahil Mehta, Aditya Choudhary, Gayathri Petluri, Vivek Lal

**Affiliations:** Department of Neurology, Postgraduate Institute of Medical Education and Research (PGIMER), Chandigarh, India

**Keywords:** Febrile illness, Leptosiprosis, Ataxia, Immune mediated

## Abstract

**Background:**

Leptospirosis involves nervous system in around 10-15% of the cases, the commonest presentation being aseptic meningitis. Most of the clinical features of neuroleptospirosis are due to capillary endothelial damage and vasculitis. Ataxia is an extremely uncommon manifestation of Leptospirosis occuring in <5% of cases.

**Case presentation:**

A 28 year old female from North India presented with a short febrile illness followed by an acute onset cerebellar ataxia, anemia, thrombocytopenia and transaminitis. Leptospira serology showed high titres of IgM (ELISA) and MAT (microscopic agglutination test titre >1:800) . She was treated with intravenous ceftriaxone for 14 days following which she showed marked recovery.

**Conclusion:**

The clinical features of neuroleptospirosis are varied, most of them resulting from endothelial damage and vasculitis. Immune mediated phenomenon with no structural damage is another possible mechanism leading to cerebellar ataxia. Cerebellar ataxia due to common tropical infections should be ruled out in the appropriate setting, as early institution of treatment can abate neurological morbidity.

The case report highlights the importance of identifying a reversible cause of cerebellar ataixa due to a tropical infection, possibly due to a immune mediated phenomenon, and would be of interest to both internists and neurologists.

## Background

Leptospirosis, a common zoonotic disease caused by spirochete, Leptospira interrogans involves nervous system in around 10-15% of the cases, the commonest being aseptic meningitis [1, 2]. Cerebellar ataxia is a rare manifestation of Leptospirosis [2]. We report a case of 28 year old female from North India who presented with pancerebellar ataxia following a febrile illness.

## Case presentation

A 28 years old female without any comorbidities presented in the month of September (just after the rainy season in North India), with complaints of high grade fever for 10 days,18 days prior to admission. 5 days after subsidence of fever, she developed gait ataxia with tremulousness of hands. The following day, she had an episode of generalized tonic clonic seizure followed by altered sensorium. On examination, she was conscious and oriented to time, place and person. Neurological examination revealed broken horizontal pursuits and hypermetric saccades, pancerebellar signs in form of gait ataxia,scanning speech, bilateral dysmetria and intention tremor,dysdiadochokinesia, rebound phenomena and impaired heel knee shin test.

**Fig. 1:**
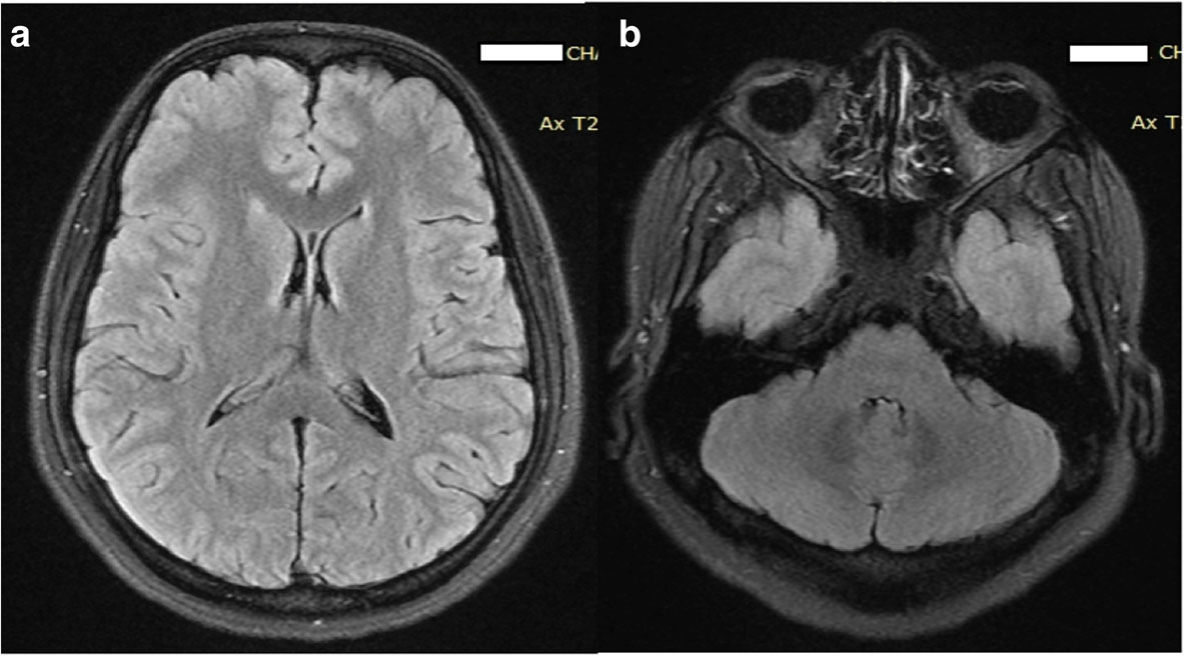
MRI images with FLAIR sequence showing normal cerebellum

On investigating, her CSF analysis was normal (no cells; normal sugar and proteins) and the cranial contrast MRI was also normal (Fig. 1). Her hemogram showed a low hemoglobin (9.4 g/dl), thrombocytopenia (platelets 86,000) with normal leucocyte count. Serum biochemistry revealed deranged liver function tests (T. bilirubin -1.9 mg/dl,Alanine aminotransferase (AST -78 U/L and Aspartate aminotransferase (ALT -88 U/L). Her renal function test (serum urea,creatinine), viral markers (HBs Ag detection and Anti HCV antibodies), Malaria work up (pLDH and anti HRP2 antibodies by Rapid Diagnostic Test, Direct microscopy with thick and thin smears) and Widal test were negative. Dengue infection was ruled out with rapid chromatographic immunoassay for NS 1 antigen and IgG and IgM antibodies. Ultrasound abdomen showed presence of fatty liver. During the hospital stay, she showed a further rise in her serum transaminases (AST -391 U/L and AST -389 U/L). Serum Leptospira serology showed high titres of IgM (ELISA-SD Bioline) and MAT (microscopic agglutination test;serovar canicola, RMRC, Port Blair,India) titre >1:800. Diagnosis of Leptospira infection was made using modified Faine’s criteria (7 - clinical, 5 - epidemiological and 15 - positive serology) [3].

She was administered intravenous ceftriaxone for 14 days. On 4th day, her anemia, thrombocytopenia and transaminitis started improving. At 1 month follow up, she had marked recovery of ataxia and she could walk independently.

## Discussion

Leptospirosis is a zoonotic disease with a worldwide prevalance and protean clinical manifestations. Humans are the secondary host who are usually infected after exposure to contaminated water, urine, blood or tissue from infected rodents. The incubation period is 1-2 weeks.

It is an important cause of acute febrile illness in China, the Indian subcontinent, Southeast Asia, Africa, South America and Central America where malaria, typhoid and dengue are also common [4].

Its clinical features range from asymptomatic infection to life threatening Weil’s syndrome. Usually a biphasic illness, theinitial leptospiremic phase may last for 3-7 days followed by an immune phase lasting 4-30 days. Leptospiremic phase is characterized by visceral involvement which can involve the liver, kidneys, hematological and respiratory system. Neurological involvement is attributed to the immune response of the body against the organism [5].

Neuro-leptospirosis occurs in around 10-15% of patients, [1] aseptic meningitis being the commonest neurological manifestation [2]. Myeloradiculopathy, myelopathy, Guillain Barre Syndrome, meningoencephalitis, intracerebral hemorrhage, tremor and rigidity have also been reported in litertature [6]. Cerebellar involvement is unusual, seen in 3-5% of the cases [5]. Prognosis in Neuro-leptospirosis is generally good but altered sensorium and seizures herald a worse prognosis [2].

Our patient presented likely in the immune leptospiremic phase with predominant pancerebellar dysfunction and improved remarkably without any sequelae. The molecular mechanisms by which spirochetes interact with cellular barriers and the chain of events involved in leptospira meningitis and other leptospirosis-related neurological phenomena remain unknown [7].

Pathologic studies have shown that most of the clinical features of neuro-leptospirosis are due to capillary endothelial damage and vasculitis [8]. Gross changes include exudates, leptomeningeal edema, brain and spinal cord congestion, and hemorrhage while pathological correlates are perivascular round cell infiltration of small and medium sized blood vessels along with patchy demyelination [9]. In our patient, no changes were seen in contrast enhanced MRI and a possible immune mediated cerebellar dysfunction, similar to that seen in autoimmune and paraneoplastic cerebellar involvement is postulated which responded to treatment of primary infection.

## Conclusions

In conclusion, Leptospirosis should be considered in the differential of postinfectious cerebellar ataxia in appropriate setting i.e., rainy season and those with a history of exposure to risk factors for leptospirosis, with or without multiorgan dysfunction.

## Abbreviations

ALT: Alanine transaminase
AST: Aspartate transaminase
CSF: Cerebrospinal fluid
ELISA: Enzyme linked immunosorbent assay
MAT: Macroscopic agglutination test
MRI: Magnetic resonance imaging
